# Know Your Client and Know Your Team: A Complexity Inspired Approach to Understanding Safe Transitions in Care

**DOI:** 10.1155/2013/305705

**Published:** 2013-11-20

**Authors:** Deborah Tregunno

**Affiliations:** School of Nursing, Faculty of Health Sciences, Queen's University, Cataraqui Building, 92 Barrie Street, Kingston, ON, Canada K7L 3N6

## Abstract

*Background*. Transitions in care are one of the most important and challenging client safety issues in healthcare. This project was undertaken to gain insight into the practice setting realities for nurses and other health care providers as they manage increasingly complex care transitions across multiple settings. *Methods*. The Appreciative Inquiry approach was used to guide interviews with sixty-six healthcare providers from a variety of practice settings. Data was collected on participants' experience of exceptional care transitions and opportunities for improving care transitions. *Results*. Nurses and other healthcare providers need to know three things to ensure safe care transitions: (1) know your client; (2) know your team on both sides of the transfer; and (3) know the resources your client needs and how to get them. Three themes describe successful care transitions, including flexible structures; independence and teamwork; and client and provider focus. *Conclusion*. Nurses often operate at the margins of acceptable performance, and flexibility with regulation and standards is often required in complex sociotechnical work like care transitions. Priority needs to be given to creating conditions where nurses and other healthcare providers are free to creatively engage and respond in ways that will optimize safe care transitions.

## 1. Introduction

Transitions in care are arguably one of the most important and challenging safety issues in healthcare today. Transitions in care involve the transfer of duties and accountabilities from one person, or group of people, to others. Transitions are a complex business that require a high degree of context-specific coordination and communication among different people with different backgrounds and skills. Consider one family's experience with transitions in care.

*Two years ago, as the time was approaching for my father to be discharged after emergency hip reconstruction surgery, and three months of non-weight bearing rehabilitation, my mother became increasingly concerned about his impending discharge. In the weeks leading up to his discharge, I had several meetings with his social worker to discuss my mother's reluctance to take my father home and her concern about his ability to once again take care of his colostomy. The social worker arranged for a number of things to happen, prior to his discharge, which enabled a smooth transition to home. A home safety assessment was conducted and bath bars and toilet seat supports were installed a week in advance of his expected date of discharge. Arrangements were made for him to attend a day hospital three times a week, including the provision of specialized transportation services. A personal care worker was arranged to bath dad three days a week and the nurses provided him with up-dated colostomy products to facilitate self-management. My mother had time to adjust to his return home and my dad thrived when he got there. *

*Just last week, my mother was discharged from the same hospital after spending three weeks in rehabilitation for a minor stroke. The day before her discharge I was still trying to meet with her social worker (i.e., a different social worker than the one who cared for my dad); several attempts to reach her by telephone and pager were unsuccessful. On one of my daily visits, I met mom's physiotherapist, who told me to adjust my dad's old walker for mom when she got home. I never did talk with the social worker, or the occupational therapist, or the family physician. I saw nurses, who never seemed to know anything about an expected date of discharge. I was surprised when the social worker called me the day before mom's discharge to ask me what time I was planning on picking her up the next day. We were given a discharge summary and mom was told to take it to her family doctor within a month. We were also given a list of her prescriptions. Only when I inquired about homecare was I told that a home safety assessment had been requested, but they were not sure if one would be provided. The transition to home was very difficult for us, and a week later we are still waiting for a home safety assessment and for someone to assist her with activities of daily living. She is becoming increasingly agitated and her condition is deteriorating. *

Why were these experiences so different? Is the difference extraordinary? Can we attribute the differences to the unique needs of each client? Can we attribute them to differences in the way healthcare providers responded to each client's needs? How can we improve care transitions and improve client outcomes? Do the existing regulatory standards of practice for nurses adequately address safety critical issues in relation to transitions in care? In this paper, we report the findings of a study undertaken with one of Canada's provincial regulatory colleges to develop a greater understanding of the kinds of regulatory requirements needed to support safe care transitions.

Three assumptions guided this work: (1) the fundamental aim of any transition in care is the transfer of high-quality clinical information between health care providers and a clear understanding by each healthcare provider about who is responsible for which aspects of the client's care; (2) regulation is one element in the web of complex interprofessional relationships and safe handoff communication; and (3) professional regulation sets the standards for skills, knowledge, and behavior for their members. We begin the paper with a review of the literature to gain a better understanding of the current knowledge related to safe transitions in care. We then consider the way in which a traditional and complexity lens helps us develop a fuller understanding of the way in which clients and families can have such dramatically different healthcare encounters, such as that illustrated in the story told above. Next, we describe the complexity informed method used to generate a rich account of client transitions from nurses, physicians, and pharmacists currently engaged in direct client care in a variety of settings in the Canadian province. Following this, we examine regulatory requirements for safe client handovers, illuminating the tension between traditional and complexity informed approaches to professional standards of practice. We conclude with recommendations to improve safe care transitions.

## 2. Literature on Transitions in Care

The literature reflects a number of terms that are applied to transitions in care. For instance, “handoffs,” “transfer of accountability,” and “handover” are terms used to describe the transfer of information and the accountability for care. Terms such as sign-over, sign-off, and sign-out are used to describe handoffs between on-call physicians. Specific to nursing, terms such as “shift change,” “change-over,” and “report” are used to mark the end of a time period and to transfer the accountability for care to the next [1]. Regardless of the nomenclature, a client handoff involves the transfer of rights, duties, and obligations from one person, or group of people, to another [2]. Communication at the time of handover should result in a clear understanding about who is responsible for which aspects of the client's care [3]. 

The evidence tells us that major gaps in care occur during these critical exchanges [4, 5]. Frequent misunderstandings, errors, and lost information are related to higher rates of unexpected readmission, failure to adhere to care plans, delays or duplication of tests/treatments, delays in obtaining consents and authorizations, inappropriate use of medication, and potentially negative clinical outcomes [6–9]. In addition, several operational challenges, such as frequent interruptions, too much noise, and lack of space, have a negative impact on care transitions. Increased client acuity results in stress related to insufficient staff time to complete the client handover [2, 10–13]. 

Despite the reality that many clients are discharged from health care settings quicker and sicker, requiring complex treatment protocols from multiple providers across multiple settings, until recently, little attention has been paid to the role professional standards of practice and safe outcomes. Overall, there is a dearth of empirical evidence that considers the link between regulatory standards of practice and safe transitions in care. Yet, to have a positive impact on the quality of care, regulatory standards of practice have to be contextually relevant, evidence based, and keep pace with the rapidly changing health care environment [14]. 

## 3. Complexity: A Different Path for Transitions in Care

A complexity lens helps nurses and other health professionals to develop a fuller understanding of the ways in which people and processes of care are interconnected, emergent and how relationship-rich networks and shared sense-making enable them to manage uncertainty more effectively [15]. Newcomers to complexity science may imagine a picture of utter confusion when they hear someone refer to healthcare as “chaotic” or “complex.” For students of complexity science, however, chaos is not about utter confusion, but rather about the existence of patterns where we thought there were none. Unlike traditional models of science, which are cast in the form of linear and proportional relationships between cause and effect, complexity models are nonlinear, and events are related within degrees of proportionality. In other words, in linear models, there is one cause for one effect. In nonlinear models, there is more than one cause for an effect and more than one effect with a cause [16]. 

From a traditional, linear perspective, the roles and responsibilities for each healthcare provider involved in care transitions are viewed as centrally structured through each discipline's professional standards of practice. There is little room for diversity and local adaptation. In contrast, from a complexity perspective, healthcare providers respond to their own particular local context, producing diversity of agent behaviours and client and family experiences. Our example illustrates how healthcare providers interact with others according to their own evolved principles of local interaction. No individual agent, or group, determines the principles of local interaction. Complexity theory reminds us that healthcare providers enable and constrain each other in local interactions, and these patterns of interaction constitute control and order. And because complex systems interact with other complex systems, tension and paradox are a common theme—seemingly opposing forces of competition and cooperation can work together in ways that can never be fully predicted or resolved. Whereas traditional scientific thinking assumes all issues can be resolved, complexity theory is comfortable with and values inherent tension between different parts of the system. Linear perspectives are based on “either-or” propositions; complexity perspectives are based on an understanding of “both.” The integration of complexity and traditional approaches to understanding processes of care during transition points will help nurses develop more comprehensive understanding of their work environment and client and family needs. 

## 4. Appreciative Inquiry: A Complexity Informed Method

The Appreciative Inquiry (AI) approach was taken to gain insight into current practices associated with care transitions and to develop insight into whether existing regulatory standards of practice for care transitions provide a sufficient underpinning to clinical practice. AI is a developmental approach to change that asks, “what is working well around here and how do we build on what is working well?” AI is based on the assumption that in every group or organization, something works well. Developed by David Cooperrider and colleagues [17] at Case Western University, AI involves the art of asking questions in ways that strengthen a system's capacity to apprehend, anticipate, and exploit procedures and processes that are working exceptionally well. Four generic AI processes were incorporated into design and execution of this study, including (1) using the positive as the focus of inquiry; (2) inquiring into exceptionally positive moments; (3) sharing the stories and identifying life-giving forces; and (4) creating shared images of a preferred future.

Relationships are vital in AI and help make sense of emerging patterns across multiple settings and roles, and so we structured the project in a way that all members of the project committee (i.e., ten representatives from the three regulatory agencies that partnered to conduct the inquiry) were directly involved in collecting and analyzing research data. At the outset of the project, we held a one-day workshop focused on AI philosophical approach and data collection methods. Subsequently, the process for data collection required each member of the project team to contact between 8 and 12 individuals from various practice settings and to invite these individuals to be interviewed either in person or on the phone. The sample was opportunistic, and a standardized AI guide was used for the interviews. Interviews lasted between thirty and sixty minutes and took place between the months of February and May 2009. Regularly scheduled teleconferences were conducted with the project team during data collection phase to address any questions and/or concerns. Each member of the project team summarized their interviews and transcribed their notes using a standardized format (approximately three pages per interview). Interview questions are provided in Box 1. 

 Content analysis was used with the data. We began our analysis by reading through the interview notes several times to comprehend their essential features. Preliminary analysis of the data involved open coding to generate a range of key themes that emerged from the data. The initial codes were then organized into provisional categories to build a coding framework divided into major themes and subthemes [18]. The author and a research assistant conducted all aspects of the coding, first independently and then we compared and discussed categories until consensus was reached. Subsequently, we engaged the project team in a one-day workshop to review and validate preliminary findings and explore implications for each profession's regulatory framework. Sixty-six interviews were conducted with a wide range of health care providers (e.g., nurses, physicians, pharmacists, social workers, physio- and occupational therapy, and pastoral care) from a variety of practice settings (e.g., acute care, long-term care, palliative care, mental health, and corrections). The number of participants from each jurisdiction is provided in Table 1. The study protocol received approval by Toronto's York University office of research ethics. 

## 5. Findings

Overall, study participants emphasized the fact that clients and families are most vulnerable when they transition between health care providers and between organizations, and we heard that nurses and other healthcare providers need to know three things to ensure safe care transitions, including (1) know your client; (2) know your team on both sides of the transfer; and (3) know the resources your client needs and how to get them. Transitions need to be timely, provide comprehensive client and family information, be customized to client needs, involve in-person exchange of information, and provide opportunities for ongoing dialogue as required. Clients are placed at even more risk when there are many interruptions during the exchange of information, when there are insufficient human resources to conduct the transfer of information, when the transfer is unplanned and unprepared, when there is a lack of respect between providers, and when there is a lack of understanding about issues of client privacy and confidentiality. 

Three key themes emerged from the interviews in response to our questions regarding successful client transfers, including (1) flexible structures; (2) independence and teamwork; and (3) client- and provider-focused care. We describe each theme here with examples from the “best” transfer experiences. 

### 5.1. Theme 1: Flexible Structures

This theme speaks of the process of client transitions, highlighting tension between the need for well-defined structures for successful transitions and the need to be flexible in order to meet unique client needs. At their best, client transitions were described as a structured process comprising several stages, including prehandoff planning, movement of the client, and posthandoff followup. They involve in-person reciprocal exchange of client information, including comprehensive data on the client's history, medications, and diagnostic testing results. Verbal report prior to physically receiving the client helps the team prepare for the client's arrival, and written documentation helps to decrease reliance on memory, thus enhancing the accuracy of the client's data. Advanced notice of a transition is especially important when clients have complex clinical needs and are being discharged between acute and homecare settings.

*During a home visit, the community nurse determined that the client needed to be assessed in the emergency department (ED). First, the community nurse provided a verbal report to the ED triage nurse telling her why she was sending the client to the ED. She also sent written documentation to the ED in the event that to a different nurse than the one to whom she provided the verbal report to ended up assessing the client. The written documentation also provided a detailed list of the correct dosages of the client's medication. (Community)*

Participants also told us that successful transitions involve a high degree of flexibility, or professional latitude. Specifically, several participants spoke about how they stretch standard operating procedures in order to assist clients, a situation which happens frequently with handoffs between institutional and home settings. Participants spoke of situations in which the hospital provided supplies and equipment for discharged clients until arrangements could be made with community providers. This flexibility contributes to successful handoffs while building a sense of good will between health care professionals working in hospital and community.

*This palliative care client was being discharged home and was prescribed IV mixture to be administered by an epidural which needed laminar flow compounding and several days to order ingredients. There was insufficient time to arrange for this medication from a community pharmacy, so the client was sent home with sufficient meds to cover the gap. This was not official hospital policy; the communication and arrangements were made through the hospital pharmacist. (Pediatrics) *

### 5.2. Theme 2: Independence and Teamwork

This theme speaks of independent and collaborative roles health care practitioners play in relation to successful handoffs. Specifically, participants discussed the importance of knowing their independent roles and responsibilities in any given situation, while respecting the skills and knowledge of teams of providers from different health care sectors and organizations. Participants stressed the importance of mutual respect when negotiating across multiple organizational and team boundaries while remaining focused on complex client needs.

*My role [as a nurse] in transferring accountability for the client is to make sure that the doctor's orders are correct, the charting is up to date, the time and date is set for transfer and to ensure that the receiving institution knows that the client is being transferred. (Acute Care)*

*Everyone knew their role really well, especially the forensic team. They made it [the handoff] a great experience because the team was very fluid and every member contributed to the efficiency of the team (Mental Health and Corrections)*

*This transfer would have been even better if there was a greater understanding of the role of health workers and parish nurses within the community, and more willingness to share health information with client consent. (Long-Term Care)*

Participants also identified the importance of collaboration between multiple healthcare teams and organizations. Collaboration is particularly important in situations of conflicting client goals, changing condition, and complex needs. In addition, several participants suggested that transfers need to be seen as a seamless continuum of care rather than independent points at which one kind of care is concluded and another starts. The following describes a situation in which multiple health and social services providers collaborated to improve the situation for one client who was living in substandard conditions:

*The individual had mental health issues and the family found it difficult to care for the individual, to the extent that they were neglectful. Efforts to improve care his care involved partnership of various organizations, including coordinated police and emergency medical services who removed the client from the unsafe environment. When presented to emergency room, the link between the his community and acute care case manager ensured sharing of his history and current situation, which facilitated an expedited review of his case by the capacity board. While the change in his care took a month of planning, it was an extremely positive experience for all involved due to teamwork and in the end, the client was in a “better place.” (Mental Health and Corrections)*

### 5.3. Theme 3: Client and Provider Focus

The third theme speaks of the information needs of clients, families, and members of the healthcare team. Clients are especially vulnerable during times of transfer and a successful handoff is measured by the degree to which the client is informed, and comfortable, the extent to which the family is informed and the degree to which staff are confident they have met client and family care needs. Specifically, study participants spoke to the importance of making sure that the client and their family members understand the reasons for the transfer and what they can expect from healthcare providers in the “new” healthcare setting. As illustrated by this participant, family members play a critical role in ensuring that client information has been shared with and between health care providers, including between nurses at shift change.

*Nurse going off shift brought the oncoming nurse to the bedside, introduced her to us and included us in a portion of her report as ID band and fluid levels checked. As a spouse, I had an opportunity to provide input regarding my husband's limitations and needs. I felt much more confident/comfortable with care provided by on-coming nurse, and my husbands' needs were better met on that shift—unfortunately this information did not seem to be passed on to subsequent shifts. (Acute Care) *

In addition, participants told us that transfers from institutional to home care are more successful when family members are involved in planning and when they have time to obtain needed supplies and equipment. This is especially true in the case of pediatric care when family members need to learn new ways of caring for their child, or when family members are entering long-term care.

*I want to share my experience of the transfer of my husband from medical floor to long-term care. Nurses on the unit came to meet us prior the transfer. Found out preferences/needs/wants plan of care had been shared ahead of time. Found out areas of chief concern, including the need to give rescue meds quickly when shortness of breath started. Decision to transfer was made based on bed availability and palliative care teams identification that my husband's needs were not being met on the medical floor. Nurses gave me a tour and showed me what was available and opportunities for me to discuss my role as a contributor to my husband's care. Were allowed to go out on pre-planned pass and when we returned, even though it was a different team, they knew our story and our preferences. (Long-Term Care)*

Study participants also spoke of the importance of transferring complete and accurate information between all members of the sending and receiving healthcare team. As stated above, successful transfer involves the exchange of comprehensive client information, including the client's medical history, a summary of the current situation, medications, test results, and those pending. Participants told us that they were more confident about their ability to provide safe care when they receive accurate, complete, and appropriate information from other team members. In addition, they spoke of the importance of trusting the providers from whom they are receiving the information.

*The positive part of the experience was the verbal report received as well as the phone call prior to transferring the client to the critical care unit that seemed to help everyone be comfortable with the admission. The RN who took the report was able to ask questions once the client arrived because they already had that head start in the information about the client before they arrived which allowed time for the RN to use critical thinking and come up with appropriate probing questions. (Acute Care) *

## 6. Discussion

To goal of this project was twofold: to gain insight into the practice setting realities for nurses and other health care providers as they manage increasingly complex care transitions across multiple settings and with a wide variety of care providers and to determine if current regulations reflect practice setting realities. Our findings demonstrate that healthcare providers responded to client and family needs in different ways, and how the outcomes of care can be dramatically different, depending on that responsiveness. Here, we discuss three key implications of our study. 

First, participants seem to be contradicting themselves as they reinforce the importance of well-defined structures for successful transitions and the need to be flexible in order to meet unique client needs. From a traditional perspective, we heard how rules, structures, and boundaries are important elements of establishing minimum expectations of performance. We believe that when treated from a traditional mechanistic framework, structures might inadvertently impede safe transitions in care by inhibiting our ability to adapt in the dynamic, ever-changing healthcare environment. From a complexity perspective, study findings alert us to the fact that successful care transitions, and safe client and family outcomes, depend on the ways in which individual healthcare providers learn from each other and respond effectively to novel situations as they arise. We heard of situations that, when facing exceptional situations, individual healthcare providers worked hard to overcome difficult dynamics and organizational policies that affect safe care transitions. In reality, the need for structures and flexibility coexists. Standard operating procedures are critically important to establish general goals and boundaries, while at the same time, the ability to explore, act on experience, and interact and respond are key to the delivery of safe and effective care in the event of ambiguous objectives and divergent problems. 

Second, while demonstrating the importance of discipline-specific knowledge and roles, our findings also demonstrate the ways in which successful transitions in care require a high degree of collaboration and teamwork with all stakeholders, and with those who have the most to gain (or lose): clients and families. Complexity science reminds us that successful care transitions involve multiple agents who are constantly interacting and changing in response to each other, and the strength of the partnership is a key factor in achieving desired outcomes. Exemplar transitions in care illustrate how relationships between individual care providers and with clients, and family members, also known as clinical microsystems [19], are the essential building block of larger systems of care. Clinical microsystems are the place where clients, families, and care teams meet, and as our findings demonstrate, are the place where interactions determine the outcome of care transitions. Consistent with what Stacey [20] refers to as complex responsive processes of relating, our finding that, at times, nurses and other healthcare workers make extraordinary responses and engage in superb teamwork to ensure safe transitions, reinforces the social nature of healthcare processes and of how safe and effective outcomes of care emerge in the social act of call and response. We suggest that nurses and other healthcare professionals take different paths, depending on whether they use traditional and/or complexity informed approaches, to manage care transitions. Do they use a for-granted approach when they interact with clients and with each other, seeing themselves as one person in a web of constrained and constraining relationships, not fully responsible for outcomes of care? or do they fully engage with each client, making sense of their need and the opportunities to collaborate with other team members to become agents of possibility and transformation? 

Third, with respect to regulatory implication, our findings demonstrated that while professional standards of practice provide the underpinning for safe care transitions, the use of good clinical judgment in the context of each client situation is a key to successful outcomes for clients, families, and care providers. In addition, findings suggest that it is easier to transfer clients within systems you know and to people you have worked with before, and that increased flexibility is required in a less familiar context. 

 Cook and Rasmussen [21] provide a complexity informed model of system dynamics that helps us understand the adequacy of existing standards of nursing practice and of how nurses and other health professionals uphold professional accountability while coming up with innovative responses and strategies to ensure safe transitions in care. In the “going solid” model of system dynamics, Cook and Rasmussen differentiate between safe and unsafe operating space; safe operating space is conceptualize as an envelope created by three boundaries: economic, workload, and performance. There is room within the safe operating space to analyze risk and respond to unique client needs with creative adaptation while still upholding practice standards [22]. However, nurses and other health care providers often operate at the margins of acceptable performance, and as our findings demonstrate, flexibility with regulation and standards is often required in complex sociotechnical work to make the system more efficient and adaptive to changing circumstances. The dynamic modeling of care transitions raises the fundamental question of how tightly regulated nurses and other healthcare professionals should be so as not to cause a trap of overregulation, which inevitably leads to more safety violations because of the tendency of standards to restrict behaviours [22]. Given the complex and context-specific nature of transitions, and the importance of clinical judgment for client safety outcomes, we concluded that a regulatory standard of practice, designed specifically for care transitions is not necessary, nor would it likely be meaningful. 

## 7. Limitations

The present study was limited to one Canadian province and the results may, to some extent, reflect contextual factors that are not shared by other national and international jurisdictions. In addition, because this study is unique in its use of appreciative inquiry as a method of research, findings may be biased toward an optimistic view of care transitions. However, we adopted this approach because we were most interested in hearing about how nurses and other health providers contribute to safe care transitions and learning about ways to support exemplary care. Members of the study team who were new to AI expressed concerns about the positive approach at the outset of the project; however, by the end of the project, these same individuals endorsed its use because the approach allowed us to build on current strengths and avoid the trap of only focusing on deficiencies. Moreover, the project provided an excellent and unique opportunity for nurses and other health professionals to gain insight into each others' practice environments and explore ways to uphold professional accountability in exceptional situations where innovations are required to ensure safe transitions in care. 

## 8. Conclusion

 Examples of positive and successful transitions demonstrated universal features across settings and healthcare providers, including the involvement of the client and family in decision making and planning, comprehensive and concise client information, opportunity for questions and followup by client and family as well as health care providers, time for planning and availability of staff to execute the transfer, and interprofessional and interorganizational collaboration. Our findings demonstrated that exemplar transitions in care are laden with paradoxes, or the coexistence of apparent opposites, including flexible structures; independence and teamwork; and client and provider focus. To ensure safe and effective care transitions, nurses and other healthcare professionals need to accept the paradoxes as a normal part of their practice environment and they need to engage in relationship-rich networks which foster creativity and responsiveness. 

All regulatory bodies establish expectations through policies, position statements, practice standards, guidelines and/or other documents, for how members do what they do in an effective, safe, and ethical manner. Viewed through a complexity lens, we see how the social act of call and response can lead to improved client and family outcomes, and that flexibility with regulation and standards is often required in complex sociotechnical work to make the care more efficient and adaptive to changing circumstances. We conclude that the development and introduction of new regulations holds the risk of constraining professionals' responsiveness at the point of care, thereby threatening client safety outcomes. Rather than focusing on new standards of practice, priority needs to be given to creating conditions where nurses and other healthcare providers are free to creatively engage and respond in ways that will optimize safe care transitions. 

## Figures and Tables

**Box 1:**
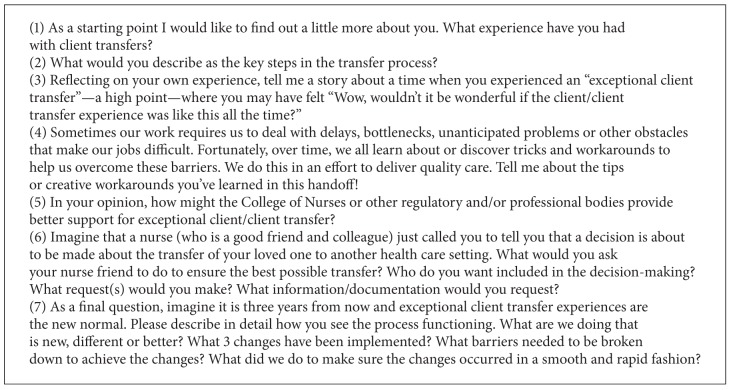
Appreciative Inquiry interview guide.

**Table 1:** Participant distribution by healthcare profession and jurisdiction.

Jurisdiction	Healthcare professional						Total
	Nurse (RN)	Pharmacist	Physicians	Social worker	Physio therapy	Chaplain	
Acute care	5	2	1	1	1		10
Community care	8	0	0		1		9
Palliative care	7	2	2	1	0	1	13
Mental health and corrections	5	2	1	2	2	0	12
Pediatrics	8	1	1	1			11
Long-term care	7	2	1	1			10

Total	40	9	6	6	4	1	66
